# Editorial: Image-guided particle therapy

**DOI:** 10.3389/fonc.2023.1175511

**Published:** 2023-04-04

**Authors:** Heng Li, Marco Durante, Oliver Jäkel, Lin Kong, Lanchun Lu

**Affiliations:** ^1^ The Johns Hopkins Hospital, Johns Hopkins Medicine, Baltimore, MD, United States; ^2^ GSI Helmholtz Center for Heavy Ion Research, Helmholtz Association of German Research Centres (HZ), Darmstadt, Germany; ^3^ Department of Medical Physics in Radiation Oncology, German Cancer Research Center (DKFZ), Heidelberg, Germany; ^4^ Department of Radiation Oncology, Shanghai Proton and Heavy Ion Center (SPHIC), Shanghai, China; ^5^ Department of Radiation Oncology, The Ohio State University, Columbus, OH, United States

**Keywords:** image guidance, particle therapy, radiation oncology, ion beam radiotherapy, proton therapy

Particle beam (e.g., Proton and Carbon) therapy has become a popular treatment modality in the field of radiation therapy for cancer treatments (1). Compared with photon beams, particle beams could administer a therapeutic dose to the tumor while limiting the dose to the surrounding healthy tissues or critical organs, thanks to the distinct depth dose distribution of the particle beam, including the characteristic Bragg Peak. Particle therapy has thus become a unique and important cancer treatment modality, with more and more particle therapy facilities have been opened or are going to be opened for clinical practice around the world. However, building a particle therapy facility costs over an order of magnitude higher than a photon therapy facility. The challenges for particle therapy are in three major areas (2) (1): image guidance (2); radiobiology (3); beam configuration and delivery. This edition of Frontiers in Oncology consists of 11 manuscripts (including 8 original research papers and 3 reviews) that cover a broad spectrum of topics associated with image-guided particle therapy.

Algranati and Strigari performed a mini-review on imaging strategies in proton therapy for thoracic tumors. Imaging is essential in a clinic’s proton beam treatment workflow: simulation, planning, setup, adaption, monitoring, and delivery. Waltenberger et al. investigated the role of ^18^F-FET PET in high-grade glioma (HCG) patients. Analyzing ^18^F-FET-PET data from a total of 76 patients, the authors found that ^18^F-FET-PET imaging might assist in prognostic stratification of HCG patients, as high SUV max value is linked to worse prognosis and unfavorable whole-blood transcriptome liquid biopsy readouts. ^18^F-FET-PET on gross tumor volume definition for particle radiotherapy should be further evaluated. The authors, therefore, suggested that larger, prospective studies were warranted.

Range verification, in addition to patient positioning, is crucial to the accurate delivery of particle beam dose. Several image modalities, including PET, CBCT, and proton radiography, were investigated in this edition for this purpose. Boscolo et al. investigated enhancing the PET signal, which could be used for range verification, with radioactive ion beams for particle therapy. The Biomedical Applications of Radioactive ion Beams (BARB) project aims to treat mice tumors with radioactive ion beams (^11^C and ^15^O). The current study showed the system’s design and plan comparison that suggests RIB could lead to margin reduction and sparing of the optical nerve in more than 50% of the patients. Schneider et al., on the other hand, reported the combination of proton radiography and irradiation for high-precision radiotherapy of small animals. The authors developed a preclinical experimental setup for small animal brain proton irradiation using proton radiography with a flat panel detector that achieved a lateral target position accuracy of <0.26 mm. Moglioni et al. analyzed patient data acquired with an in-beam Positron Emission Tomography (PET) scanner, which enabled *in-vivo* range monitoring in proton and carbon therapy treatments at the National Center of Oncological Hadrontherapy (CNAO), Italy. Eight patients treated with protons were analyzed in the work, where the author found the overall standard deviation in activity range difference to be 2.5 mm. The author also found a larger observed range difference with PET for patients with morphological changes than those without. Li et al. evaluated the proton range uncertainty with daily cone-beam CT of phantom and patient data. They found that the range uncertainties from CBCT images were larger than from CT but could still be used for daily dose validation in selected patients. Reidel et al. studied the image artifacts in CT, visibility in daily imaging, and dose perturbations in proton beams resulting from different fiducial markers that were composed of different materials, including gold, platinum, and ZrO_2_. Notably, high-resolution CMOS pixel sensors were used to quantify fluence perturbations due to fiducial marks in proton beams. The authors found that the perturbations were reduced for small and low-density marks of mass lower than 6mg. The authors thus suggested that the trade-off between image quality and dose perturbation needs to be considered in the fiducial selection for setup.

Advance in imaging also enables advanced treatment delivery techniques requiring precise treatment delivery. Zhou et al. reviewed methods of robust angle selection in particle therapy in various disease sites. The authors noted that with technological development, including improving imaging technologies in particle RT, robust angle selection would become more precise and individualized, improving the effectiveness of particle RT. Li et al. investigated the feasibility of incorporating linear energy transfer (LET) in spot-scanning proton arc therapy optimization. The authors demonstrated that incorporating LET into the proton arc optimization could maximize the LET distribution wherever desired inside the target while averting the high LET away from the adjacent organ at risk. Liu et al. compared the dosimetric and potential benefits of using Stereotactic body radiotherapy (SBRT) with the simultaneous integrated boost (SIB) technique for locally advanced pancreatic cancer. Analyzing 19 patients, the authors found that the tumor location could play a critical role in determining clinical benefits among different treatment modalities: two-field IMPT might be a better option for the pancreas head, whereas VAMT could offer better protection when the tumor is located in the pancreas body. Volz et al. reviewed considerations for upright particle therapy patient positioning and associated image guidance. Based on the literature review, the author found that upright patient positioning could have distinct economic and clinical benefits but also face clinical and engineering challenges in order to achieve highly accurate and stable patient positioning. The authors suggested that the advancement of upright positioning systems, including imaging systems, could bring a paradigm shift for the future of particle therapy.

In summary, the treatment’s precision and accuracy are crucial for particle therapy’s efficiency and efficacy, partly due to the need to accurately place the Bragg Peak. Imaging is critical throughout particle therapy workflow, improving targeting, dosimetry, and treatment delivery accuracy. Currently, the imaging modalities used for imaging guidance for almost all particle therapy treatments are CBCT or kV X-ray. Although CBCT and kV X-ray guidance are widely accepted image guidance techniques for radiation therapy, there are some well-known limitations for these radiation-based imaging modalities, including poor soft tissue contrast and difficulty performing online/real-time monitoring during treatments and hence the adaptive radiation therapy. Developing imaging techniques that improve soft tissue contrast, such as MRI-guided particle therapy, and real-time image guidance methods, such as PET and prompt gamma imaging, is therefore valuable. Such development would overcome the limitations of state-of-the-art radiation-based imaging guidance and add the capabilities of treatment outcome assessment and prognosis with functional image features and with the potential to reduce the treatment cost of particle therapy. Additionally, incorporating artificial intelligence (AI) in image guidance for particle therapy could further improve the treatment (3). While AI has been implemented for IGRT in some capacity, prospective studies are still required to expand these AI-related applications and realize their potential.

## Author contributions

All authors listed have made a substantial, direct, and intellectual contribution to the work and approved it for publication.
